# Supplementary material for: Structural insights into strigolactone catabolism by carboxylesterases reveal a conserved conformational regulation
Source: Nat Commun. 2024 Aug 1;15:6500. doi: 10.1038/s41467-024-50928-3 (PMC11294565; doi:10.1038/s41467-024-50928-3)
Supplement: Supplementary file 1 — Supplementary Information [file 41467_2024_50928_MOESM1_ESM.pdf]

Supplementary Information

Structural Insights into Strigolactone Catabolism by Carboxylesterases Reveal a Conserved Regulation through Conformational Dynamics

Malathy Palayam[1], Linyi Yan[1], Ugrappa Nagalakshmi[1], Amelia K. Gilio[1], David Cornu[2], François-Didier Boyer[3], Savithramma P. Dinesh-Kumar[1,4], and Nitzan Shabek[1*]

[1]Department of Plant Biology, College of Biological Sciences, University of California - Davis, Davis, CA 95616
[2]Université Paris-Saclay, CEA, CNRS, Institute for Integrative Biology of the Cell (I2BC), 91198, Gif-sur-Yvette, France
[3]Université Paris-Saclay, CNRS, Institut de Chimie des Substances Naturelles, UPR 2301, 91198, Gif-sur-Yvette, France
[4]The Genome Center, University of California - Davis, Davis, CA 95616

*Correspondence should be addressed to: nshabek@ucdavis.edu

Supplementary Figures and Datasets

Fig. S1: SL structure and sequence analysis of Arabidopsis CXEs.

Fig. S2: Sequence conservation and phylogenetic analysis of AtCXEs.

Fig. S3: Purification, crystallization and electron density maps of AtCXE15 and AtCXE20

Fig. S4: Sequence analysis of CXE15 within Brassicaceae species.

Fig. S5: Sequence analysis of CXE20 within Brassicaceae species.

Fig. S6: Comparative structure analysis of AtCXE15, AtCXE20 with plant and animal Carboxylesterases.

Fig. S7: Structural characterizations of CXE15 and CXE20 from the trajectories of molecular dynamics simulation.

Fig. S8: Schematic representation of PVX system and expression of CXE15 and CXE15$^{S169A/E271A}$ in *N. benthamiana.*

Fig. S9: Biochemical characterization of CXE15 and CXE20

Fig. S10: Mass Spectrometry characterization of CXE15^{apo}, CXE15-GR24 CXE20^{apo} and CXE20-GR24 complexes.

Fig. S11: Proposed mechanism of CXE20 based on Mass Spectrometry analysis.

Fig. S12: Uncropped Protein gels used in main and supplementary figures.

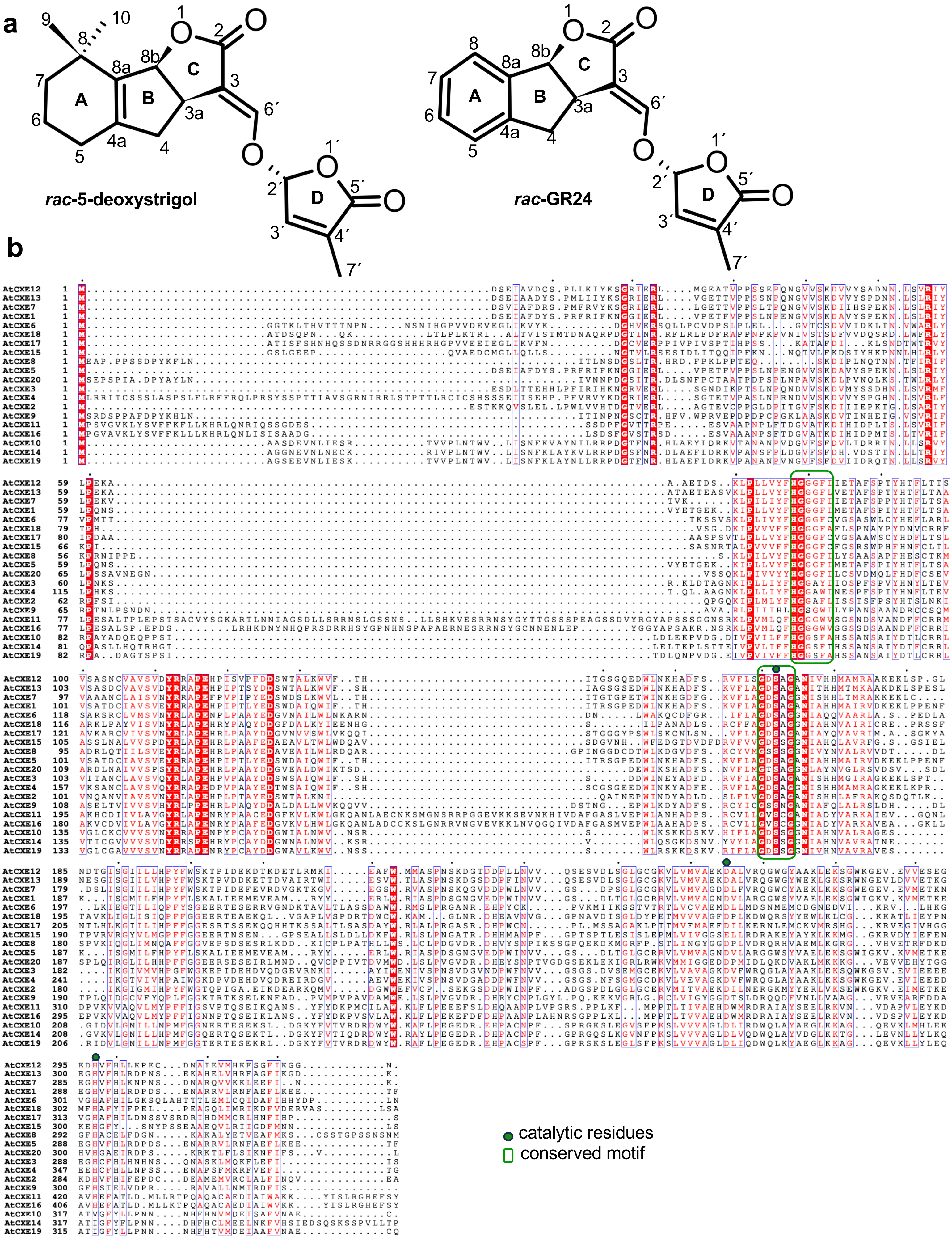
a
rac-5-deoxystrigol
rac-GR24
b
AtCXE12
AtCXE13
AtCXE7
AtCXE1
AtCXE6
AtCXE18
AtCXE17
AtCXE15
AtCXE8
AtCXE5
AtCXE20
AtCXE3
AtCXE4
AtCXE2
AtCXE9
AtCXE11
AtCXE16
AtCXE10
AtCXE14
AtCXE19
catalytic residues
conserved motif

Supplementary Figure 1. SL structure and sequence analysis of Arabidopsis CXEs. (**a-b**) Chemical structure of natural canonical strigolactone, 5-deoxystrigol (***a***) and synthetic hormone, *rac*-GR24 (***b***). Amino acids of 20 Arabidopsis thaliana carboxylesterases (AtCXEs) are aligned for comparison. Intensity of red behind the residues shows the degree of conservation. Circle in green indicates the catalytic triad and box in green indicates the conserved motifs present across CXEs.

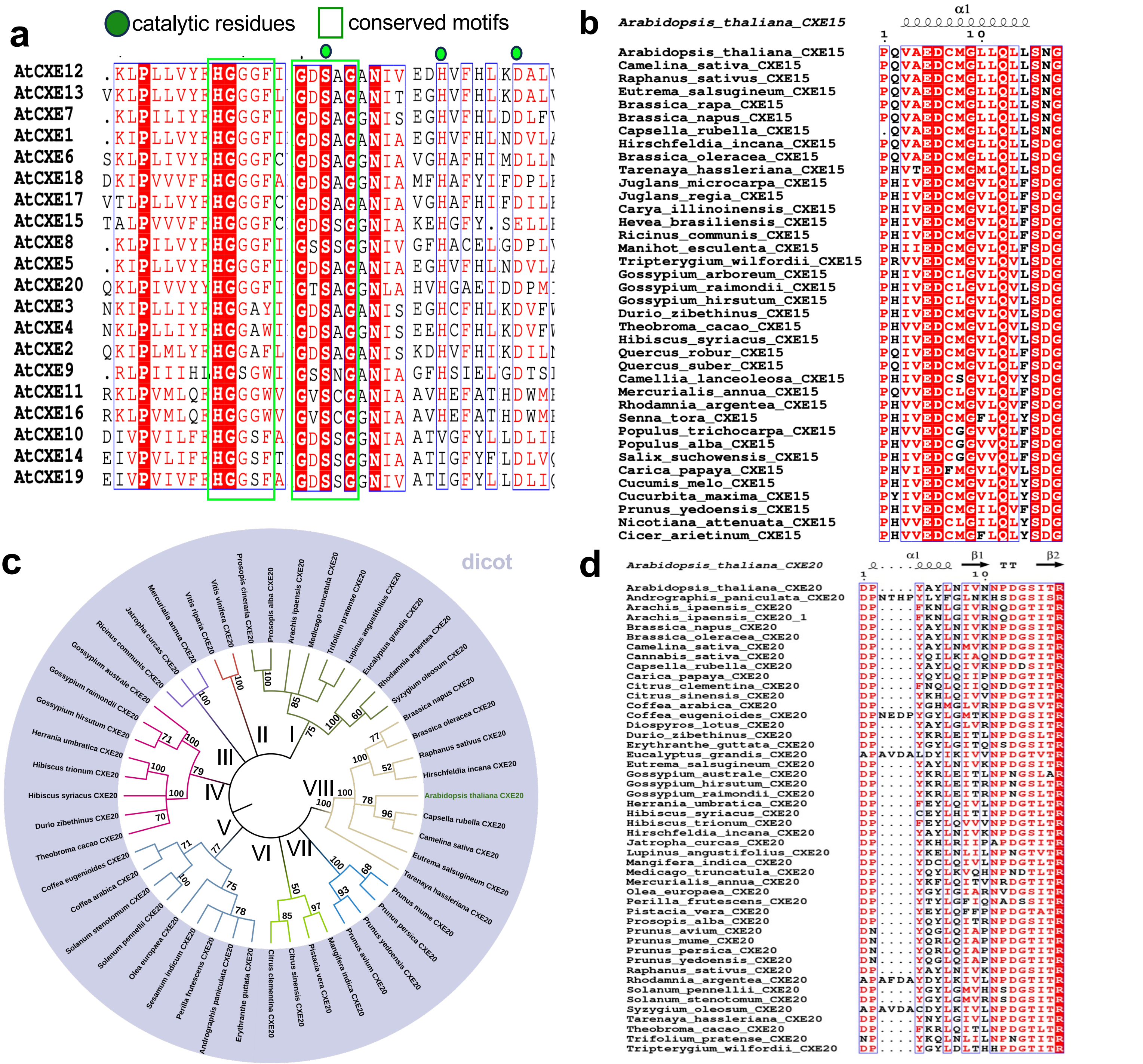

Supplementary Figure 2. Sequence conservation and phylogenetic analysis of CXEs. (**a**) Amino acids sequence alignment of 20 CXEs from *Arabidopsis thaliana (At)*. The sequence highlighted in the box represents the conserved HGGGF motif and GDSAG motif. The green circle represents the catalytic residues present across the 20 CXEs. In the naming *At* represents *Arabidopsis thaliana*. (**b** and **d**) Amino acids conservation analysis of N-terminal region of CXE15 and CXE20 from dicots. Intensity of red behind the residues shows the degree of conservation. Numbers on residues refer to position in *At*CXE15 sequence. Secondary structure of CXE15 and CXE20 are shown on top of the alignment. (**c**) Phylogenetic tree of Arabidopsis CXE20 based on the protein sequences retrieved from NCBI and constructed using MEGA11 and maximum likelihood algorithm. The values on the nodes represents the bootstrap replicates of 1000 and values greater than 50% are shown.

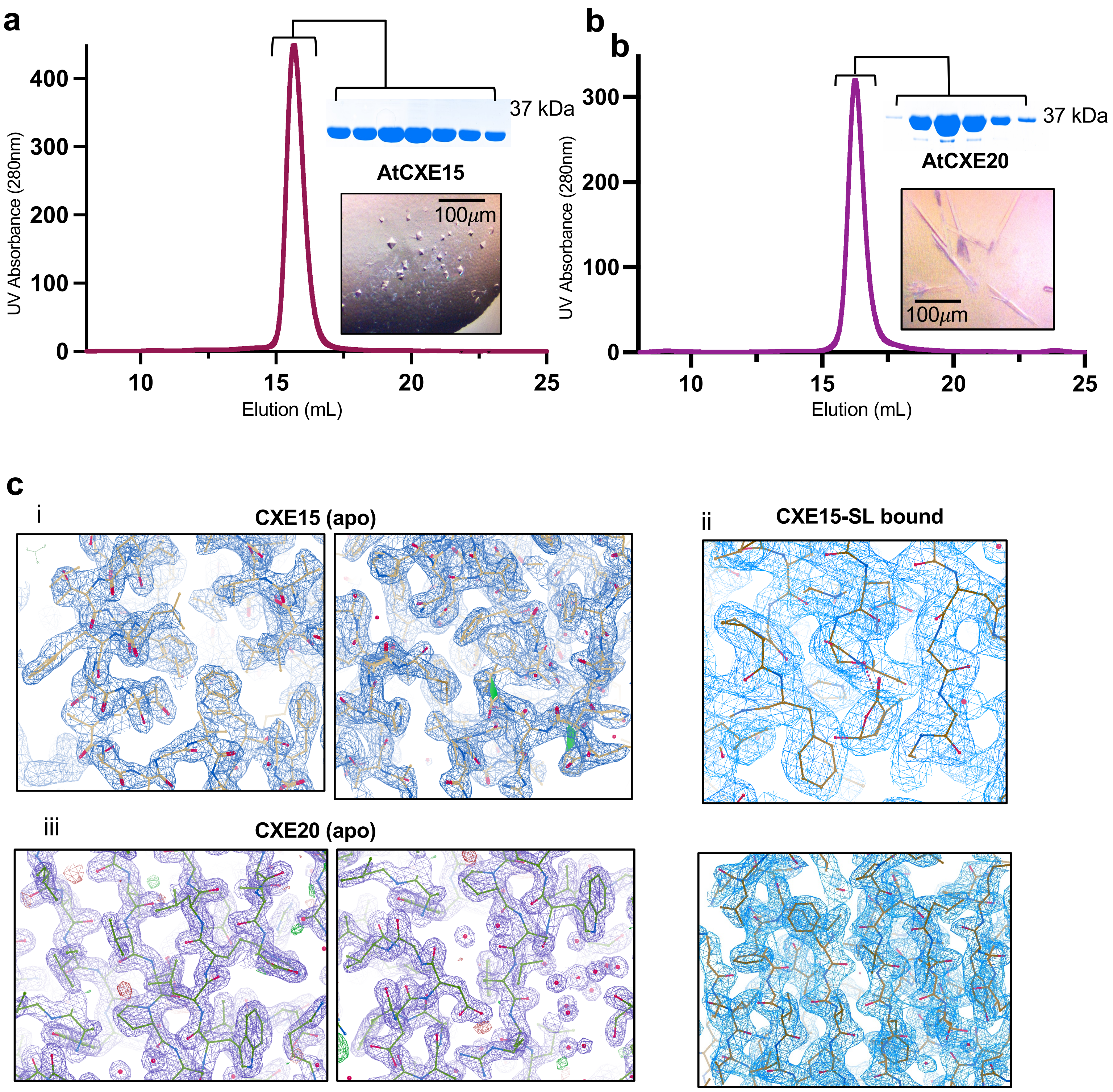

Supplementary Figure 3. Purification crystallization and electron density maps of AtCXE15 and AtCXE20. (a-b) Size exclusion analysis of purified AtCXE15 and AtCXE20 respectively. Crystals of *At*CXE15 and AtCXE20 are shown in the box. MW were estimated by biomolecule standard markers. Elution fractions were resolved by SDS-PAGE and Coomassie staining. Elution from SEC were concentrated and screened for crystallization. (**c**) Electron density and omit maps of CXE15^{apo} (i), CXE15-SL bound (ii) and CXE20^{apo} (iii) structures. Electron density and omit map is shown at 2.2σ and 1.0σ cut-off respectively.

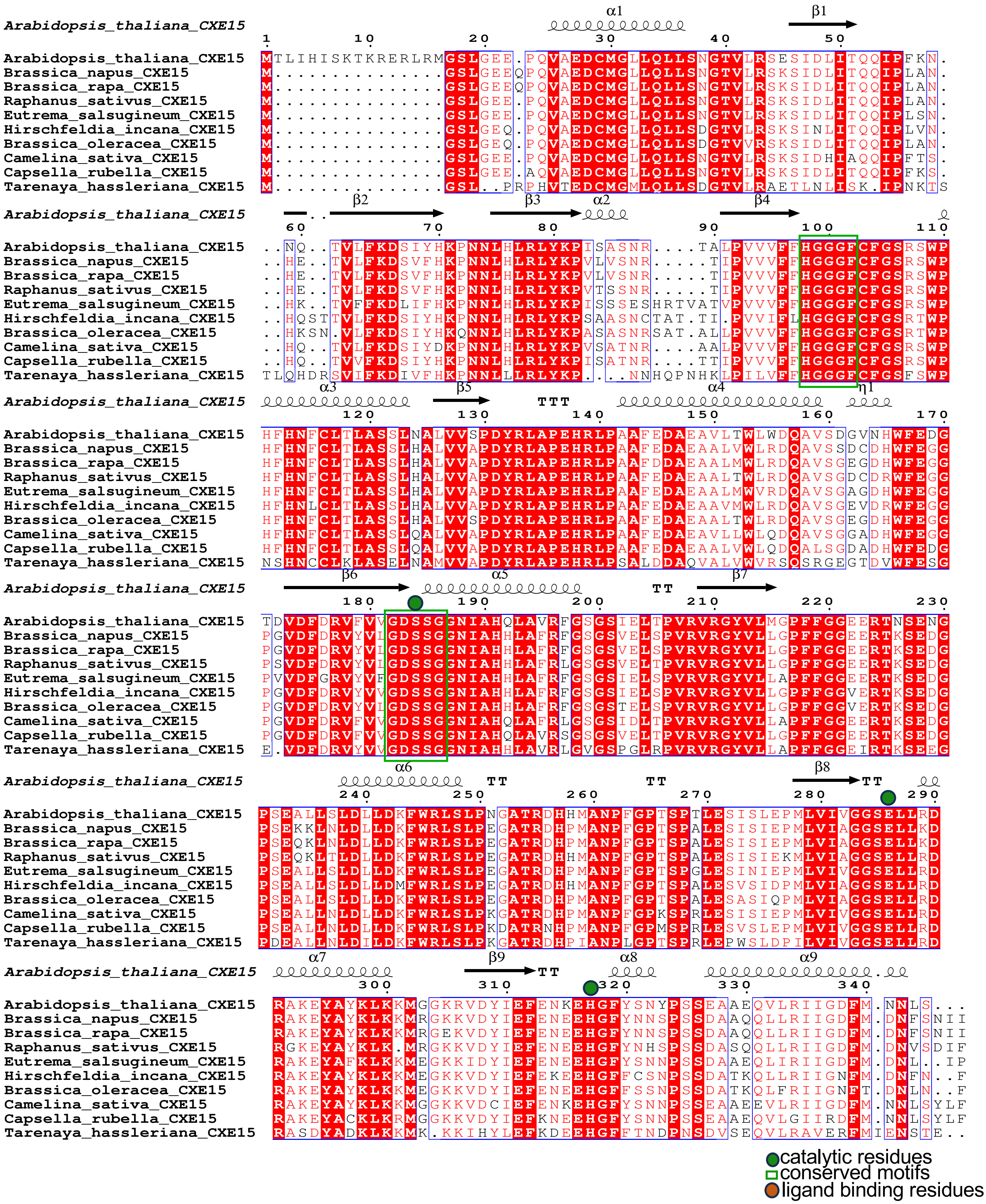

Supplementary Figure 4. Sequence analysis of CXE15 within Brassicaceae species. Amino acids of CXE15 are aligned for comparison. Intensity of red behind the residues shows the degree of conservation. Circle in green and brown indicates the catalytic triad and SL interacting residues respectively. Box in green represents the conserved motifs in CXE15. Numbers on residues refer to position in *At*CXE15 sequence. Secondary structure of CXE15 is shown on top of the alignment.

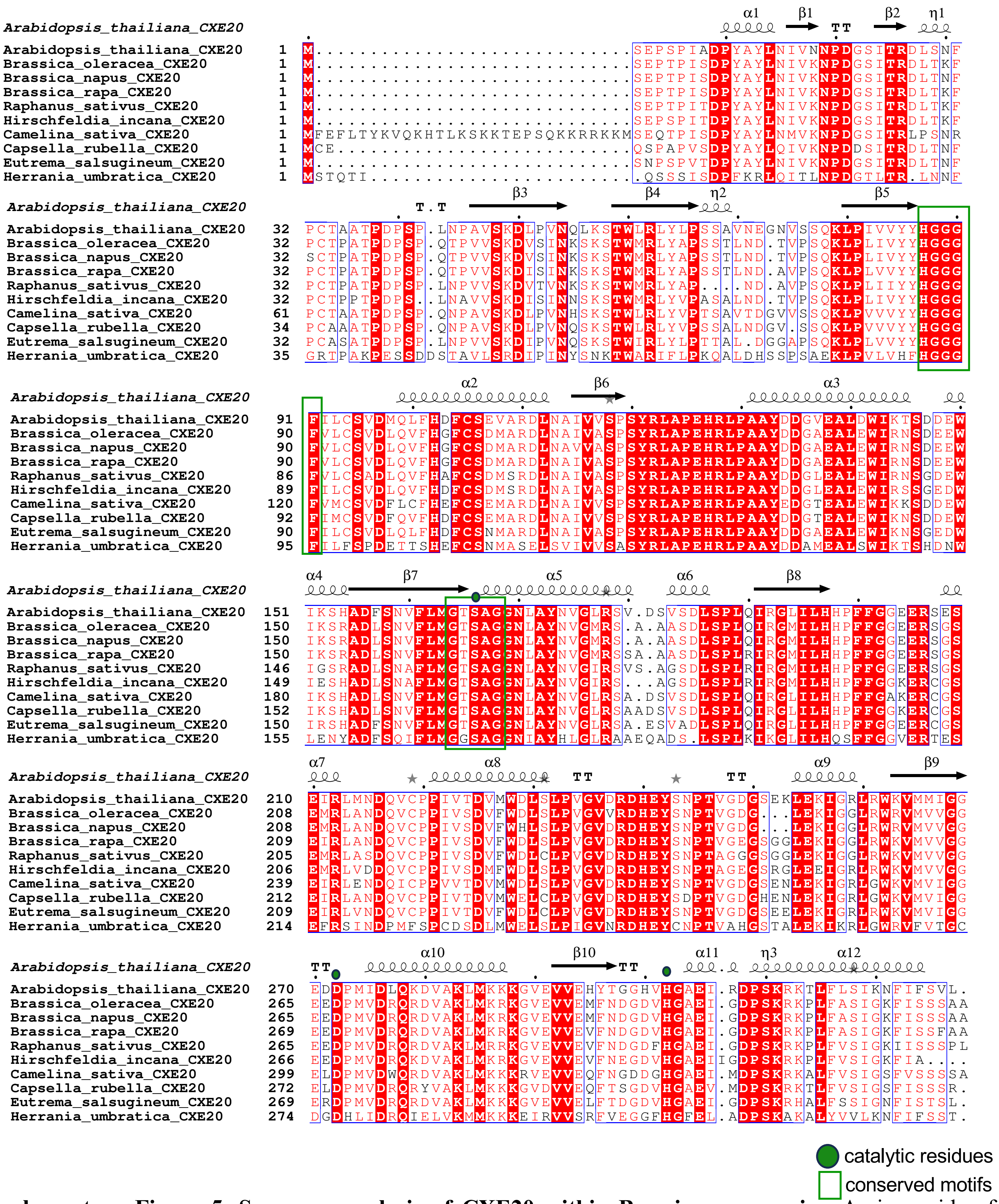

Supplementary Figure 5. Sequence analysis of CXE20 within Brassicaceae species. Amino acids of CXE20 are aligned for comparison. Intensity of red behind the residues shows the degree of conservation. Circle in green and brown indicates the catalytic triad and SL interacting residues respectively. Box in green represents the conserved motifs in CXE20. Secondary structure of CXE20 is shown on top of the alignment.

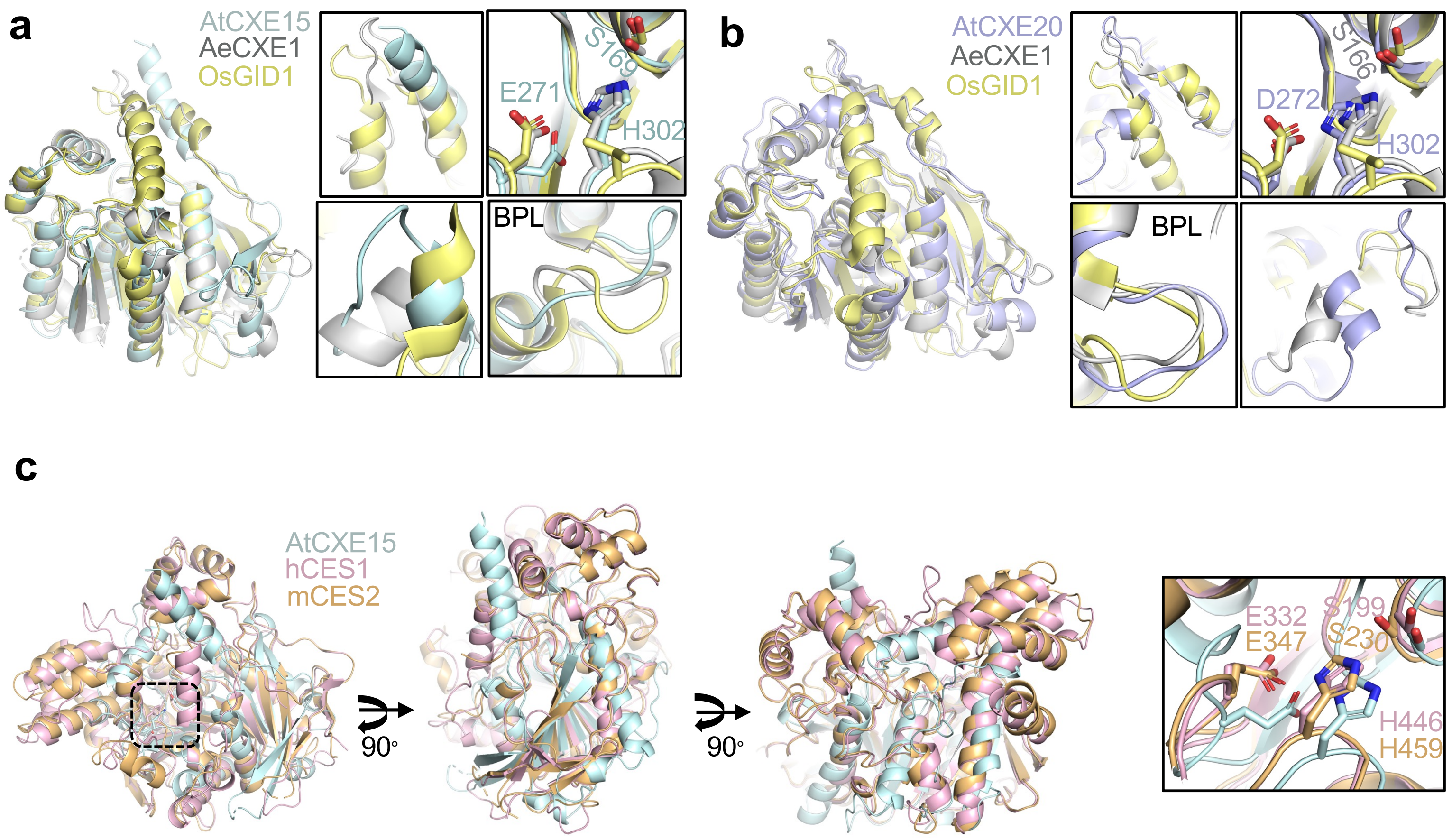

Supplementary Figure 6. Comparative structure analysis of AtCXE15 and AtCXE20 with plant and animal carboxylesterases. (**a-b**) Superposition of AtCXE15 reported here (cyan) (***a***) and AtCXE20 reported here (light purple) (***b***) with kiwi CXE1 (grey, PDB ID: 2O7R) and rice GID1 (light yellow, PDB ID: 3EBL). Close-up view of the catalytic triad (residues number refer to CXE15/20 respectively), binding pocket loop (BPL) and overall differences in their structures are shown in cartoon (right). Root mean square (RMS) deviation of 0.89Å , 0.82Å and 1.2Å, 1.9Å was measured between AtCXE15 and AtCXE20 with kiwi CXE1 and rice GID1 respectively. (**c**) Superposition of AtCXE15 (cyan) with human carboxylesterase (hCES1, PDB ID: 1MX9, light pink) and mouse carboxylesterase (mCES2, PDB ID: 8AXC, light orange). Close up view of the catalytic triad in AtCXE15, hCES1 and mCES2. RMS deviation of 14.5Å and 12.9Å was measured between CXE15 with hCES1 and mCES2 structures (right).

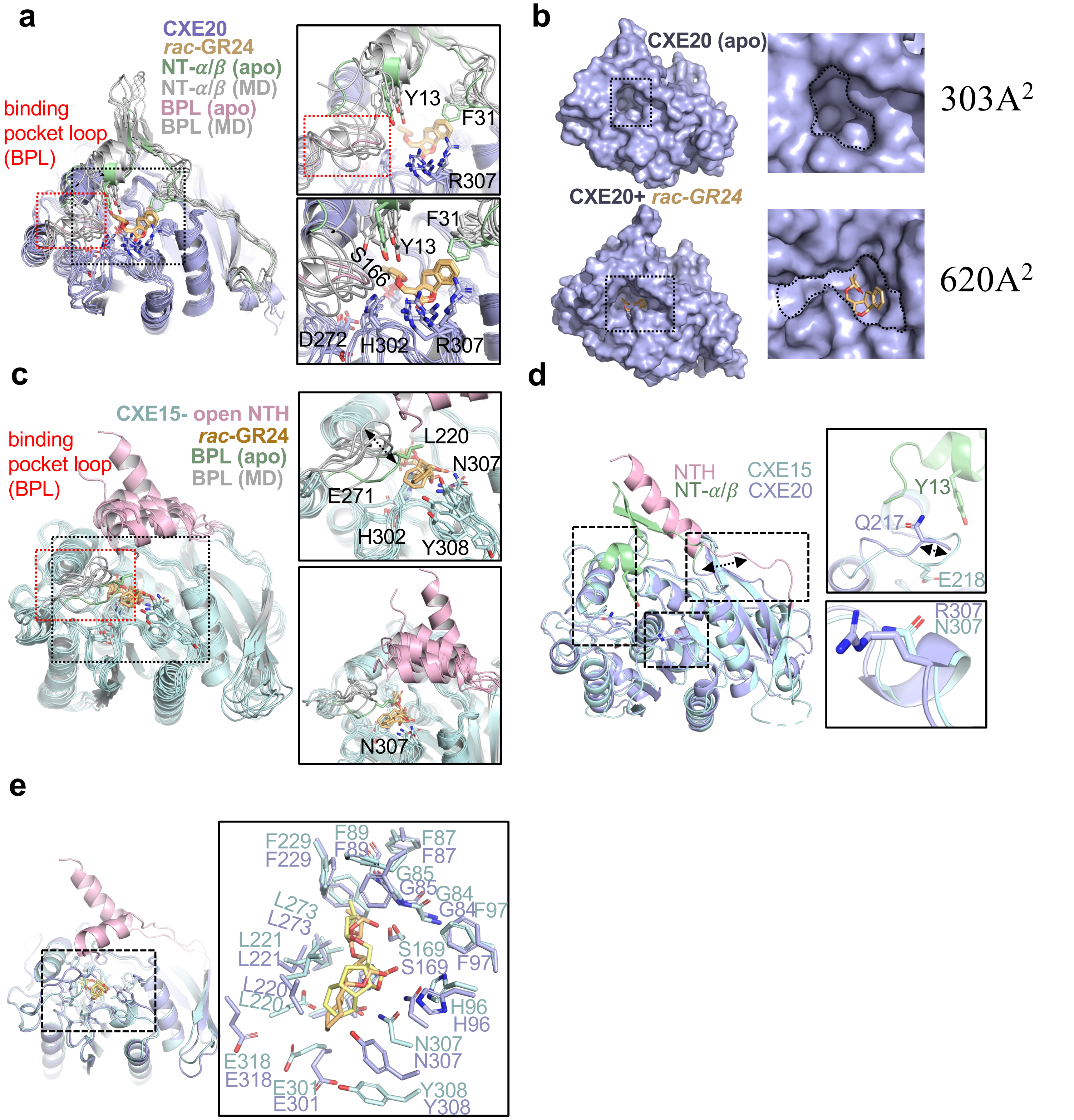
a
CXE20
rac-GR24
NT-α/β (apo)
NT-α/β (MD)
BPL (apo)
BPL (MD)
binding pocket loop (BPL)
Y13
F31
R307
F31
Y13
S166
D272
H302
R307
b
CXE20 (apo)
303A²
CXE20+ rac-GR24
620A²
c
CXE15- open NTH
rac-GR24
BPL (apo)
BPL (MD)
binding pocket loop (BPL)
L220
N307
E271
H302
Y308
N307
d
NTH
NT-α/β
CXE15
CXE20
Y13
Q217
E218
R307
N307
e
F229
F229
F89
F89
F87
F87
G85
G85
G84
F97
L273
L273
L221
L221
S169
S169
F97
L220
L220
H96
H96
N307
N307
E318
E318
E301
E301
Y308
Y308

Supplementary Figure 7. Structural characterizations of CXE15 and CXE20 from the trajectories of Molecular dynamics simulation. (**a**) comparative structural superposition of $CXE20^{apo}$ and $CXE20^{GR24}$ from the molecular trajectories (left). BPL is highlighted in red box. Close-up view of the structural superposition of BPL (BPL^{apo}, light pink and BPL^{MD} , light grey) (top-right). Close-up view of the catalytic pocket showing the dynamics of amino acid residues within the cavity (bottom-right). (**b**) Comparative structural analysis of $CXE20^{apo}$ and $CXE20^{MD}$ shown in surface representation (left). Zoom in view of the catalytic pocket of $CXE20^{apo}$ (top-right). Close-up view of the catalytic pocket of $CXE20^{MD}$ (bottom-right). (**c**) Comparative structure analysis between CXE15-open NTH (open state, light pink) and CXE15-closed NTH (closed state, light pink). The dynamics of BPL^{apo} (light green) is compared with BPL^{MD} (light grey). Close-up view of BPL showing $closed^{BPL}$ (light green) to $open^{BPL}$ (light grey) state (top-right*)*. Close-up view of NTH showing $closed^{NTH}$ (light pink) to $open^{NTH}$ (light pink) state (bottom-right*)*. (**d**) Comparative crystal structural analysis of CXE15 (pale cyan) and CXE20 (light green) (left). The NT loop connecting the core region of CXEs varies between CXE15 and CXE20. Close-up view of BPL showing the kink in CXE20 is caused by the insertion of Y13 from its NT region (top-right). Zoom in view of the amino acid variation between CXE15 and CXE20 (bottom-right). (**e**) Superposition of CXE15 (open NTH, pale cyan) and CXE15 (closed NTH, purple) bound to GR24 (left). Close-up view of the interaction of amino acid residues within the catalytic cavity in the presence of GR24 in open (light yellow) and closed state (wheat) (right).

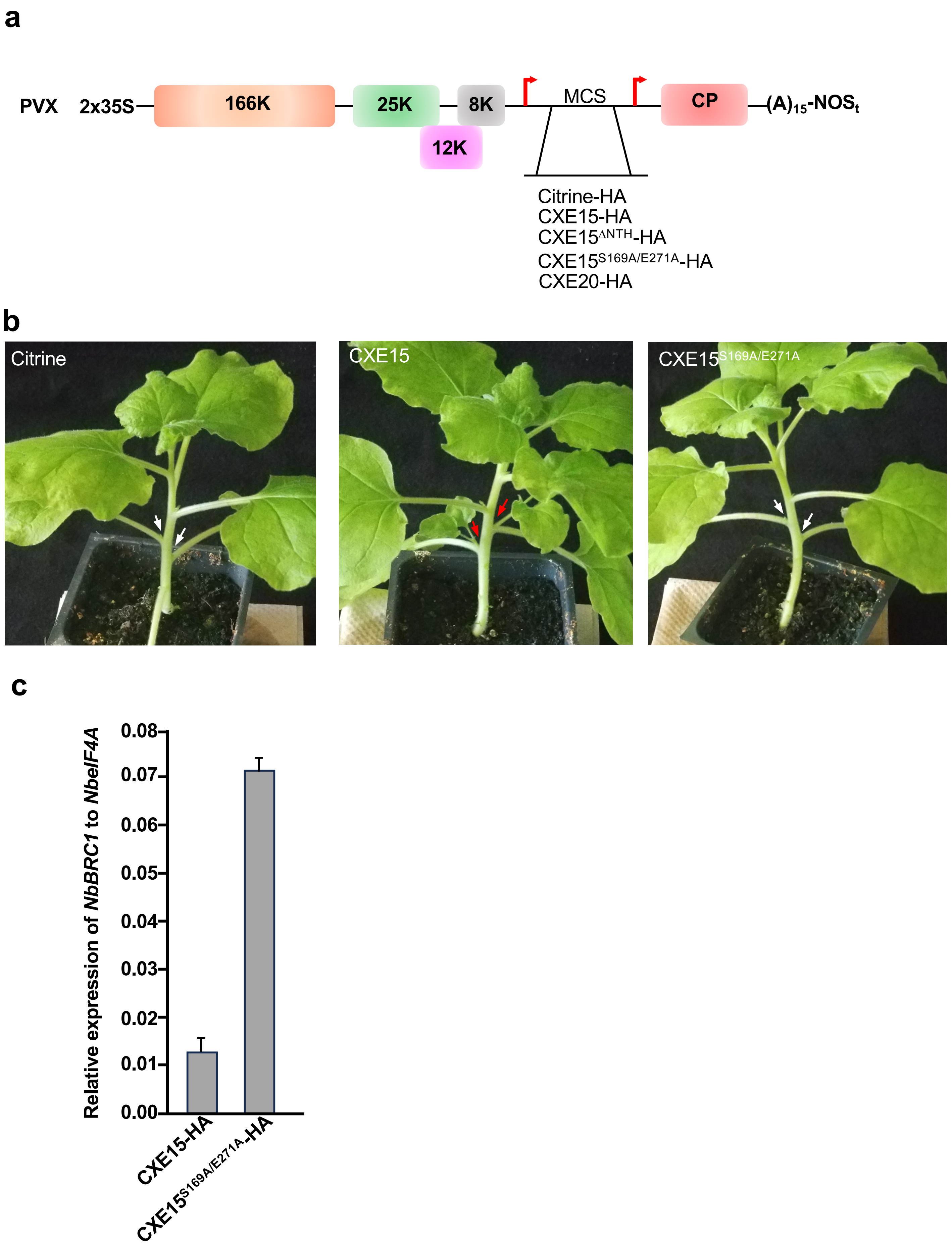
a
PVX
2x35S
166K
25K
8K
12K
MCS
CP
(A)15-NOSt
Citrine-HA
CXE15-HA
CXE15ΔNTH-HA
CXE15S169A/E271A-HA
CXE20-HA
b
Citrine
CXE15
CXE15S169A/E271A
c
Relative expression of NbBRC1 to NbeIF4A
0.08
0.07
0.06
0.05
0.04
0.03
0.02
0.01
0.00
CXE15-HA
CXE15S169A/E271A-HA

Supplementary Figure 8. Schematic of Potato Virus X system and expression of CXE15 and CXE15 mutants in *N. benthamiana.* (**a**) Full-length cDNA of PVX is under the control of a double 35S promoter from Cauliflower mosaic virus (2x35S) and nopaline synthase terminator (NOSt). Viral RNA-dependent RNA polymerase (166K), coat protein (CP), triple gene block movement proteins (25K, 12K, and 8K) and poly A (A) are depicted. CP subgenomic promoter is duplicated (red arrows) and multiple cloning sites (MCS) are engineered to express protein of interest. CXE15, CXE15$^{\Delta NTH}$, CXE15$^{S169A/E271A}$, CXE20 and citrine coding sequences were cloned into the MCS region for expression in *N. benthamiana* plants. (**b**) Agrobacterium with PVX-Citrine, PVX-CXE15, and CXE15$^{S169A/E271A}$ were infiltrated into two opposite leaves of 3-weeks old *N. benthamiana* plants. Representative photographs shown were taken two weeks post-infiltration. Experiments were repeated twice. Red arrows point to auxiliary branches and white arrows point to no auxiliary branches. (**c**) Expression of *NbBRC1* in axillary buds. Three weeks old *Nicotiana benthamiana* plant leaves were infiltrated with PVX::CXE15-HA or PVX:: CXE15$^{S169A/E271A}$. Twenty days post-infiltration, RNA extracted from the axillary bud tissue was used for qRT-PCR with *NbBRC1* specific primers. *NbeIF4A* expression was used to normalize the expression value of each sample. Data are shown as mean ±SD.

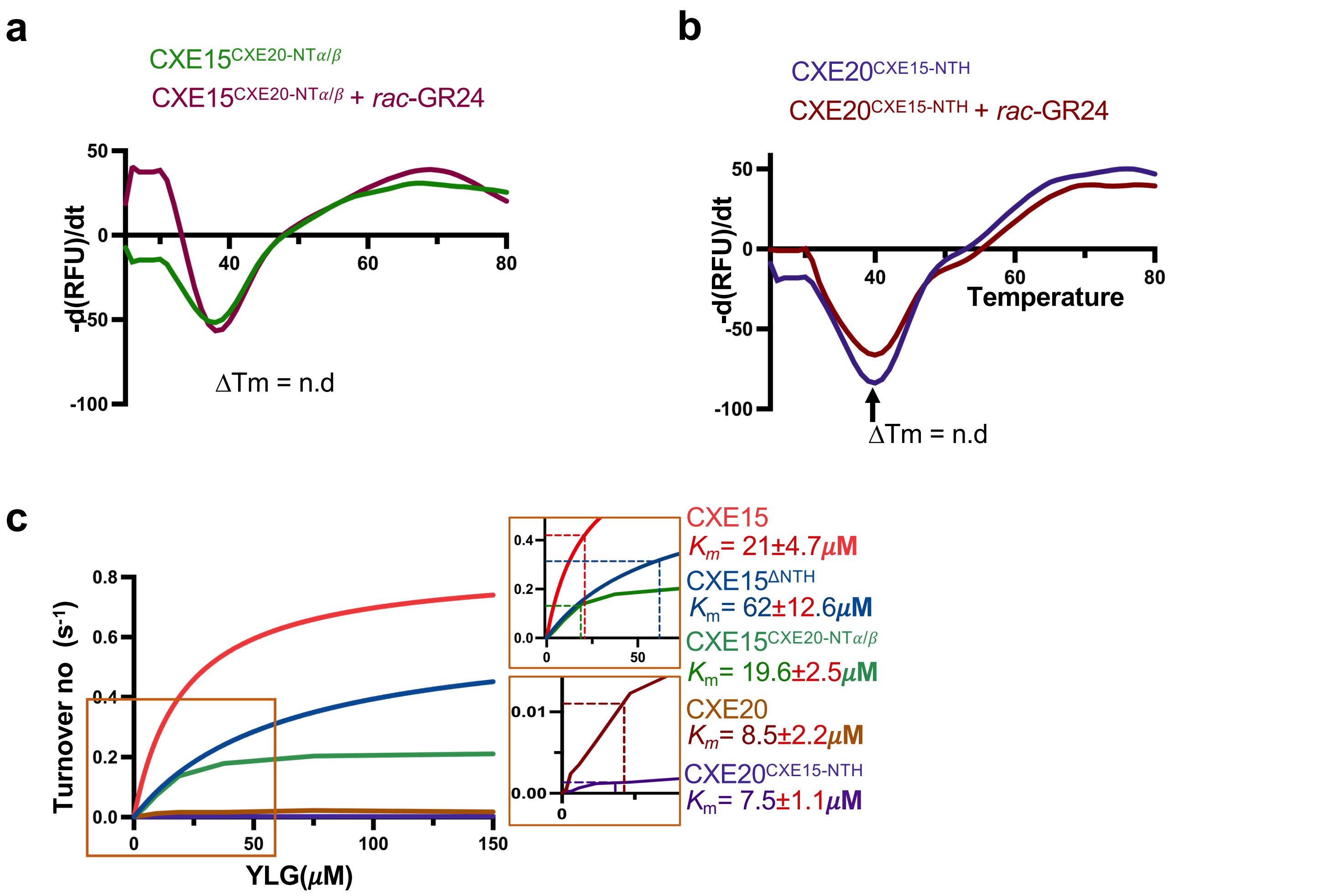

Supplementary Figure 9. Biochemical characterizations of CXE15 and CXE20. (a-b). Melting temperature curves of CXE15$^{CXE20\text{-}NT\alpha/\beta}$ (green), CXE15$^{CXE20\text{-}NT\alpha/\beta}$ with *rac*-GR24 (brown) (a) and CXE20$^{CXE15\text{-}NTH}$ (purple), CXE20$^{CXE15\text{-}NTH}$ with *rac*-GR24 (brown) determined through DSF (b). (**c**) Comparative YLG hydrolysis turnover rates of CXE15 (red), CXE15$^{\Delta NTH}$ (*blue*), CXE15$^{CXE20\text{-}NTa/b}$ (green), CXE20$^{CXE15\text{-}NTH}$ (*purple*) and CXE20 (brown) (left). Zoom in view of Michaelis constant (K_m, mM) showing the affinity of substrates (top and bottom-right).

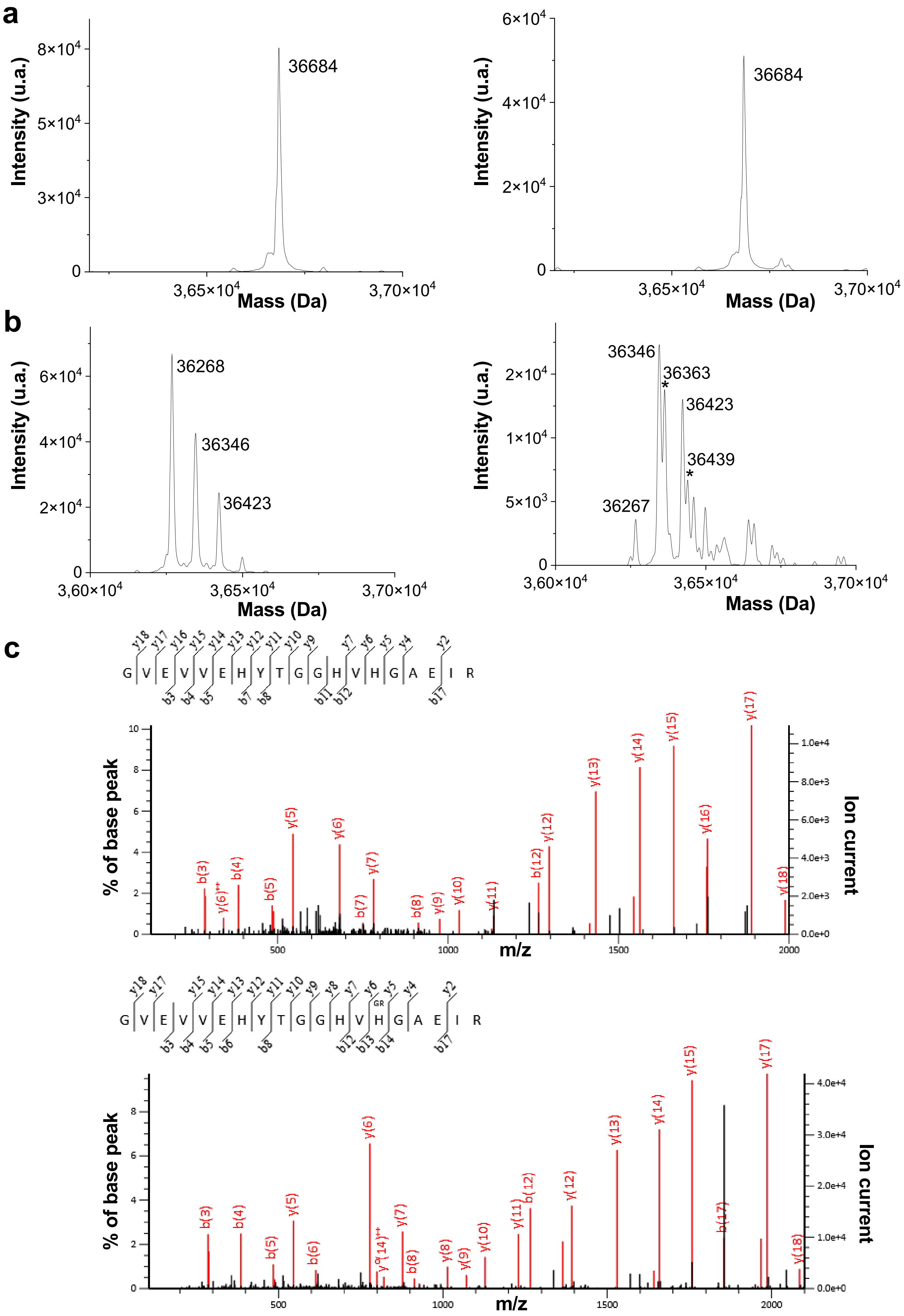
a
Intensity (u.a.)
Mass (Da)
36684
36684
b
36268
36346
36423
36346
36363
36423
36439
36267
c
G V E V V E H Y T G G H V H G A E I R
% of base peak
Ion current
m/z
G V E V V E H Y T G G H V H G A E I R
GR
% of base peak
Ion current
m/z

Supplementary Figure 10. Mass Spectrometry characterization of CXE15^{apo}, CXE15-GR24 CXE20^{apo} and CXE20-GR24 complexes. (**a**) Deconvoluted electrospray mass spectra of CXE15^{apo} (left), CXE15-*rac*-GR24 (right). (**b**) Deconvoluted electrospray mass spectra of CXE20^{apo} (left), CXE20-*rac*-GR24 (right). Peaks with an * represents CXE20 covalently bound to a *rac*-GR24. A mass increment of 96Da is measured for CXE20-*rac*-GR24 complex. (**c**) Fragmentation spectra analyses of unmodified (CXE20^{apo}) (top) and covalently modified CXE20-*rac*-GR24 complex (bottom) by nano LC-MSMS after trypsin proteolysis. Labeled peaks correspond to b and y fragments of the triple charged precursor ion. H302 amino acid modified by *rac*-GR24 is marked with GR on the sequence displayed on the top.

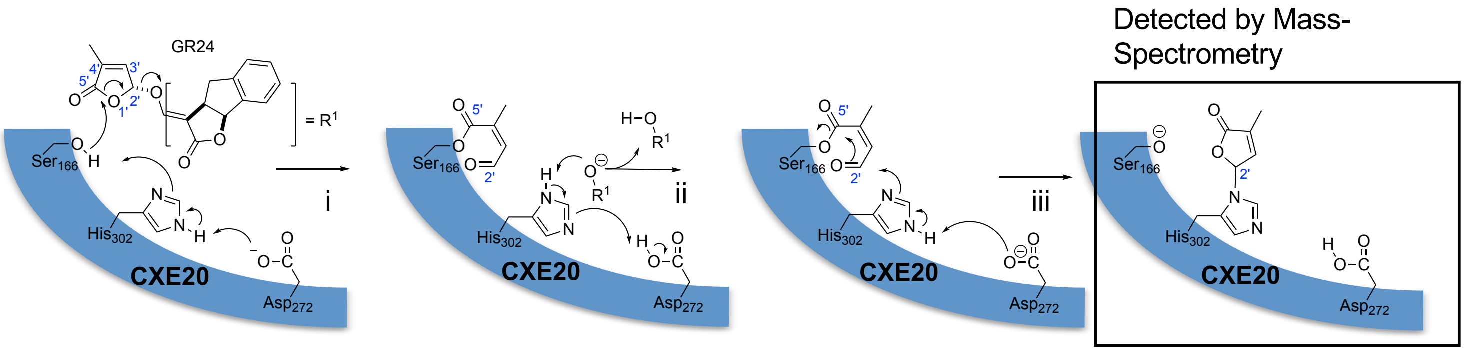

Supplementary Figure 11. Proposed mechanism of CXE20 based on Mass Spectrometry analysis. (i) Nucleophilic attack by S166 of CXE20 on the carbonyl carbon at C5′ position resulting in the formation of covalent linkage and further departure of the tricyclic ring as a leaving group. (ii-iii) The re-arrangement of proton between the leaving group-H302 and D272 results in the second nucleophilic attack at the aldehyde intermediate at the C2′ position by the nitrogen atom in H302 results in the formation of covalent bond with the D-ring of *rac*-GR24.

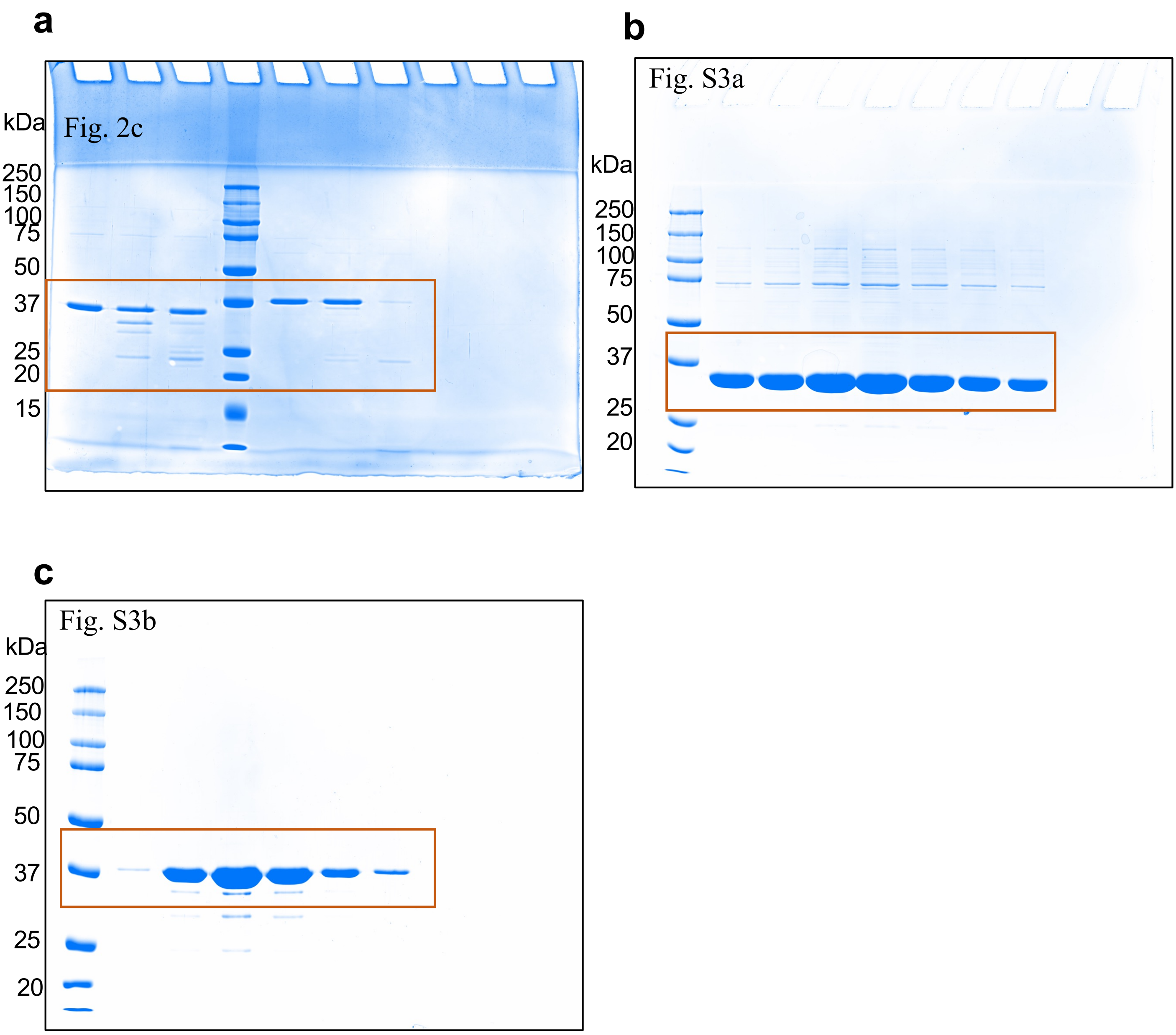

Supplementary Fig. 12 Uncropped Gels used in Main and supplementary figures. (a) Uncropped gel used for Fig.2c (b) Uncropped gel used for Fig.S3a (c) Uncropped figure used for Fig.S3c. Orange box indicates gel region used for figures. Molecular weight markers in kDa are shown in left
