# Supplementary material for: Structural insights into strigolactone catabolism by carboxylesterases reveal a conserved conformational regulation
Source: Nat Commun. 2024 Aug 1;15:6500. doi: 10.1038/s41467-024-50928-3 (PMC11294565; doi:10.1038/s41467-024-50928-3)
Supplement: Supplementary file 3 — Description of Additional Supplementary Files [file 41467_2024_50928_MOESM3_ESM.pdf]

Description of Additional Supplementary Files

Supplementary Movie Legends

Supplementary Movie 1: Molecular dynamic (MD) simulation analysis of CXE15 and CXE20 with GR24. Left panel (top: cartoon representation; bottom: surface view); the Nterminal helix (NTH) of CXE15 transitions from an open to a closed state, acting as a lid, upon binding with rac-GR24 (colored orange). The binding pocket loop (BPL) of CXE15 moves away from the catalytic pocket (S169, H302 and E271, shown in sticks presentation) to accommodate the substrate. Right panel (top: cartoon representation; bottom: surface view); the NT-αβ region of CXE20 does not undergo major open-closed transition, and the BPL of CXE20 has limited flexibility. The loop connecting NT-αβ to the catalytic core shifts away from the catalytic pocket, (S166, H302 and D272, shown in sticks), resulting in an increased pocket size. The protein structures from the molecular trajectory were saved as a single PDB file and analyzed via PyMOL v-3.0. The different states of CXE15 and CXE20 were visualized and recorded using Morph on PyMOL v-3.0.

Supplementary Data Legends

Supplementary Data 1: Amino acid sequences of Arabidopsis CXEs used for phylogenetic analyses.

Supplementary Data 2: Amino acid sequences of plant CXE15 used for phylogenetic analyses.

Supplementary Data 3: Amino acid sequences of plant CXE20 used for phylogenetic analyses.

Supplementary Data 4: Amino acid sequences of the N-terminal region of dicot CXE15 and CXE20 were used for sequence conservation analyses.
